# Expression of an extremely acidic β-1,4-glucanase from thermoacidophilic *Alicyclobacillus *sp. A4 in *Pichia pastoris *is improved by truncating the gene sequence

**DOI:** 10.1186/1475-2859-9-33

**Published:** 2010-05-14

**Authors:** Yingguo Bai, Jianshe Wang, Zhifang Zhang, Pengjun Shi, Huiying Luo, Huoqing Huang, Chunliang Luo, Bin Yao

**Affiliations:** 1 Key Laboratory for Feed Biotechnology of the Ministry of Agriculture, Feed Research Institute, Chinese Academy of Agricultural Sciences, Beijing 100081, PR China; 2 Biotechnology Research Institute, Chinese Academy of Agricultural Sciences, Beijing 100081, PR China

## Abstract

**Background:**

*Alicyclobacillus *sp. A4 is thermoacidophilic and produces many glycoside hydrolases. An extremely acidic β-1,4-glucanase (CelA4) has been isolated from *Alicyclobacillus *sp. A4 and purified. This glucanase with a molecular mass of 48.6 kDa decreases the viscosity of barley-soybean feed under simulated gastric conditions. Therefore, it has the potential to improve the nutrient bioavailability of pig feed. For the study reported herein, the full-length gene, *CelA4*, of this glucanase (CelA4) was identified using the sequences of six peptides and cloned from strain A4. The gene fragment (*CelA4*_*F*_) encoding the mature protein was expressed in *Pichia pastoris*. Sequence truncation and glycosylation were found for recombinant CelA4_F_, both of which affected the expression efficiency. The physical properties of various forms of CelA4 as they affected enzymatic activity were characterized.

**Results:**

We located the full-length 2,148-bp gene for CelA4 (*CelA4*) in the genome of *Alicyclobacillus *sp. A4. *CelA4 *encodes a 715-residue polypeptide with a calculated molecular mass of 71.64 kDa, including an N-terminal signal peptide (residues 1-39), a catalytic domain (residues 39-497), and a C-terminal threonine-rich region (residues 498-715). Its deduced amino acid sequence and that of an *Alicyclobacillus acidocaldarius *endo-β-1,4-glucanase were identical at 44% of the residue positions. When the experimental molecular mass of CelA4_F_--a recombinant protein designed to mimic the CelA4 sequence lacking the N-terminal signal peptide that had been expressed in *Pichia pastoris*--was compared with its hypothetical molecular mass, it was apparent that CelA4_F _was truncated, possibly at residue 497. An artificially truncated gene fragment (*CelA4*_*T*_) without C-terminal threonine-rich region was expressed in *P. pastoris*, and the expression efficiency of CelA4_T _was substantially greater than that of CelA4_F_. Purified CelA4_F _and CelA4_T _had similar molecular masses (~60 kDa) and enzymatic properties (optimum pH, 3.4; optimum temperature, 60°C); they were relatively stable between pH 1.2 and 8.2 at 70°C and resistant to acidic and neutral proteases. However, their molecular masses and thermostabilities differed from those of CelA4 isolated from *Alicyclobacillus *sp. A4. A deglycosylated form of CelA4 (CelA4_D_) had properties similar to that of CelA4 except that it was thermoliable at 60°C.

**Conclusions:**

Truncation during expression of CelA4_F _or artificial truncation of its gene--both of which produced a form of CelA4 lacking a threonine-rich region that includes a putative linker--increased the level of enzyme produced in comparison with that produced by cultivation of *Alicyclobacillus *sp. A4. Glycosylation increased the thermostability of CelA4. Of the four forms of CelA4 studied, CelA4_T _was produced in highest yield and had the most favorable physical properties; therefore, it has potential for use in the feed industry.

## Background

β-Glucan is the major cell-wall component of cereals such as barley, wheat, oat, and rye [1], and it can be hydrolyzed by β-glucanases. Microbial glucanases are often used in industry, including those related to waste management [2], alcohol fermentation [3], and animal feed production [4]. Several β-glucanases from the genus *Alicyclobacillus *have been identified, including two endoglucanases (CelA and CelB) [5,6], one β-1,4-glucanase (CelA4) [7], one β-1,3(4)-glucanase (Agl9A) [8], and one cellulase (CelG) [9]. All are very thermoacidophilic and have optimum activities between 45 and 60°C and pH 2.0 and 6.0.

According to the primary structures of their catalytic domains, β-1,4-glucanases have been classified as members of the glycoside hydrolase (GH) families 5, 6, 7, 8, 9, 12, 44, 45, 48, and 51 http://www.cazy.org/fam/acc_GH.html. Most of the GH 51-type glucanases are α-L-arabinofuranosidases. Only three β-glucanases, endoglucanase F precursor from *Fibrobacter succinogenes *(AAC45377) [10], endoglucanase CelB from *A. acidocaldarius *(CAD86595) [6], and a cellulase from an uncultured bacterium (CAF22222.1) [11], are GH 51-type glucanases.

We previously purified the extremely acidic GH 51-type β-1,4-glucanase, CelA4 with a molecular mass of 48.6 kDa, from the thermoacidophilic *Alicyclobacillus *sp. A4 [7]. The pH optimum of CelA4 is 2.6, it is protease resistant, and can decrease the viscosity of barley-soybean feed under simulated gastric conditions. These properties indicate that CelA4 may improve the nutrient bioavailability of pig feed. For the commercialization of CelA4, recombinant gene expression in a high-throughput fermentation system is necessary. The methylotrophic yeast, *Pichia pastoris*, is an excellent host for the heterologous expression of recombinant proteins for which expression is controlled by the alcohol oxidase 1 promoter [12]. High-cell-density fed-batch cultivation usually consists of four phases and has been widely used to improve protein expression in *P. pastoris *[13]. The purpose of the study reported herein was to obtain the gene for CelA4 and, using it, to develop a high-yield fermentation process for CelA4 in *P. pastoris*. Upon doing so, we then examined how the physical properties of the native and recombinant enzymes affected enzymatic activity and identified certain properties that affected expression efficiency.

## Results

### Identification and sequence analysis of the full-length β-1,4-glucanase gene (CelA4)

**Figure 1:**
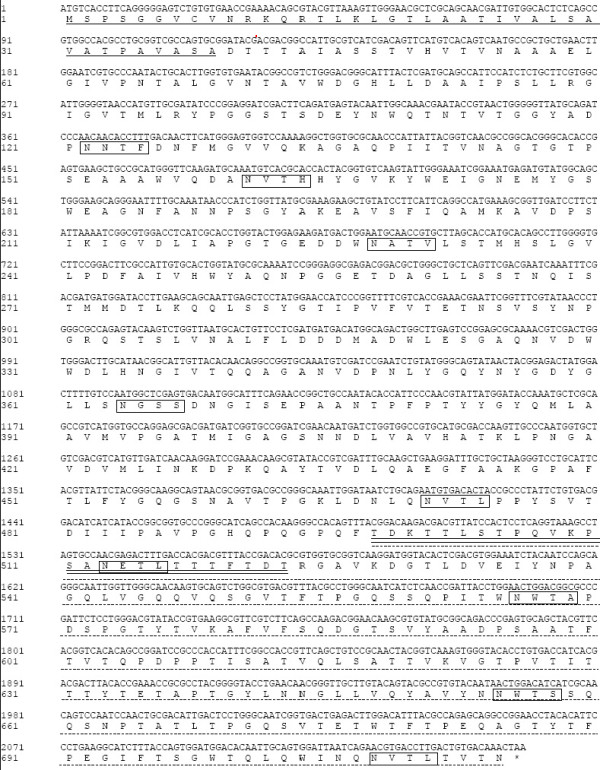
**Nucleotide and amino acid sequences of the β-1,4-glucanase, CelA4, from the thermoacidophilic *Alicyclobacillus *sp. A4**. The residues of the putative signal peptide (residues 1-39) are underlined, those of the linker region are underlined twice, and the threonine-rich region is indicated by dashed line, nine putative N-glycosylation sites are indicated by black boxes.

**Figure 2:**
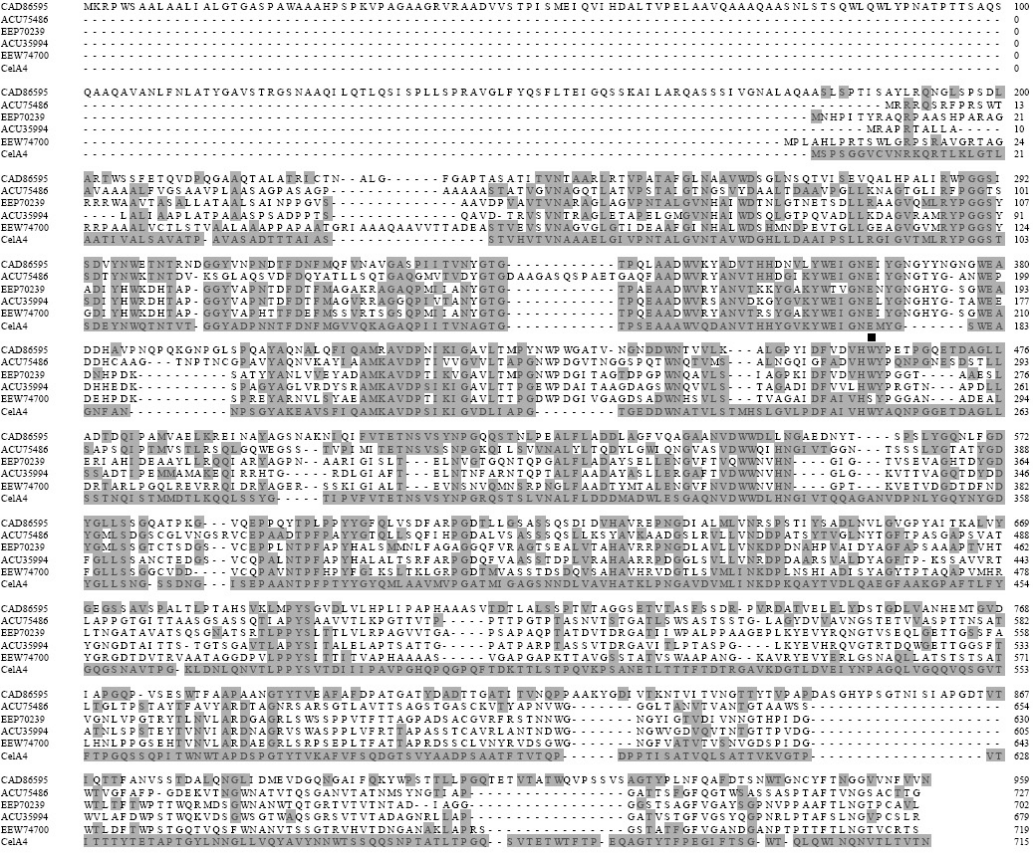
**Amino acid sequence alignment of CelA4_F _and five other glucanases**. The sequences were those of glucanases from *Alicyclobacillus acidocaldarius *DSM 446 (CAD86595), *Catenulispora acidiphila *DSM 44928 (ACU75486), *Micromonospora *sp. ATCC 39149 (EEP70239), *Actinosynnema mirum *DSM 43827 (ACU35994), and *Streptomyces flavogriseus *ATCC 33331 (EEW74700). ClustalW was used to align the sequences. Residues that are the same at all positions are indicated by solid grey boxes; the catalytic residue, Glu176, is indicated by a solid square.

We located the 2,148-bp full-length β-1,4-glucanase gene (Figure 1) (*CelA4*) in the *Alicyclobacillus *sp. A4 genome using six known peptide sequences of CelA4 as identifiers [7]. *CelA4 *encodes a 715-residue polypeptide (calculated molecular mass, 71.64 kDa), which includes an N-terminal signal peptide (residues 1-39), a catalytic domain (residues 39-497), and a C-terminal threonine-rich region (residues 498-715, 21.12% threonine). Pro, Asp, Ser, and Glu--typical linker amino acids [14]--comprise 62% of residues 498-523, and therefore this region is presumed to be a linker. The enzyme also contains nine putative N-glycosylation sites having the consensus sequence, Asn-Xaa-Thr/Ser-Zaa, where Zaa is not Pro; five of these sequences are in the catalytic region, and four are in the threonine-rich region. The deduced amino acid sequence of *CelA4 *is most similar (44% identical) to that of the GH 51 cellulase, CelB, from *A. acidocaldarius *(CAD86595) [6]. The threonine-rich regions of these two enzymes have only 28% of their residues in common, and the threonine-rich region of CelA4 has <15% sequence identity with those of other glucanases. Alignment of CelA4 with five other glucanases using ClustalW is shown in Figure 2. The sequence alignment indicated that CelA4 does not contain a carbohydrate (cellulose)-binding domain found in the four glucanase sequences of ACU75486 (residues 625-724), EEP70239 (residues 600-699), ACU35994 (residues 575-676), and EEW74700 (residues 613-716). The putative catalytic residue in CelA4, Glu176, is highly conserved in glucanases and is located within the active site as predicted by sequence alignment [15,16].

### Expression and purification of recombinant CelA4_F _in P. pastoris

**Figure 3:**
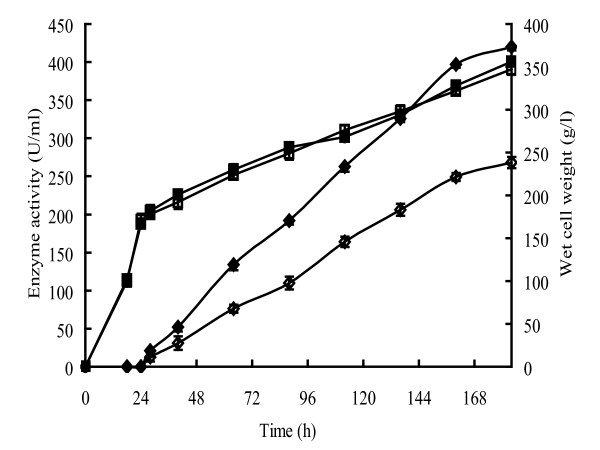
**Time courses for the appearance of β-1,4-glucanase activity and increase in biomass during fermentation**. The symbols and associated proteins are as follows: diamond stands for activity; Square stands for biomass; hollow stands for CelA4_F _and solid stands for CelA4_T_.

The gene (*CelA4*_*F*_), which encodes a form of CelA4 that lacks the N-terminal signal sequence, was cloned into the pPIC9 vector that was then transformed into *P. pastoris *competent cells. The cells were cultured and clones were isolated. The clone that had the highest β-1,4-glucanase activity after flask cultivation was selected for expression in a 3.7-L fermenter. During fermentation, the cell mass of recombinant *P. pastoris *kept increasing from phase one (~110 g/L; about 18 hours) to phase four (~350 g/L; about 184 hours) (Figure 3) (See Methods for descriptions of the cultivation phases). The β-1,4-glucanase activity in the supernatant was 268 U/mL 156 h after induction with methanol.

**Figure 4:**
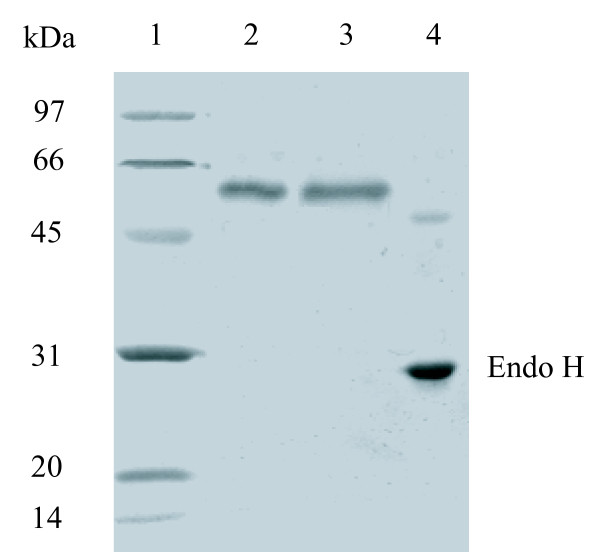
**SDS-PAGE gels of purified CelA4_F _and CelA4_T _before and after Endo H treatment**. Lanes: 1, standard protein molecular-weight markers; 2, purified CelA4_T_; 3, purified CelA4_F_; 4, purified CelA4_F _after deglycosylation with Endo H.

Recombinant CelA4_F _was purified by anion exchange chromatography. It migrated as a single band upon SDS-PAGE and had an apparent molecular mass of ~60 kDa (Figure 4), which is less than the predicted molecular mass (71.64 kDa) of CelA4_F_, but greater than that of CelA4 (48.6 kDa). Using LC-ESI-MS/MS, this band was identified as CelA4_F_. These results indicate that recombinant CelA4_F _was probably both truncated and glycosylated.

### Deglycosylation and artificial truncation of CelA4_F_

Because CelA4 has nine putative N-glycosylation sites, the observed variation in apparent molecular mass of CelA4_F _might be ascribed to glycosylation and/or truncation. After endo-β-*N*-acetylglucosaminidase H (Endo H) treatment, the protein migrated as a single band of 48 kDa upon SDS-PAGE, and thus the decrease in apparent molecular mass was ascribed to deglycosylation. The molecular mass of deglycosylated CelA4_F _(CelA4_D_) is almost the same as that of CelA4. Therefore, CelA4_F _was both glycosylated and truncated when expressed in *P. pastoris*.

According to the molecular mass comparison and sequence analysis, residue 497 was predicted to be the truncation site. CelA4_T _was expressed in *P. pastoris *as described above. The *P. pastoris *culture for CelA4_T _followed the same growth profile as for CelA4_F_. The β-1,4-glucanase activity in the supernatant of the CelA4_T _culture reached 420 U/mL (Figure 3), which was much higher than that found for the CelA4_F _culture. Purified CelA4_T _migrated as a single band upon SDS-PAGE and had a molecular mass of ~60 kDa, which is the same as found for CelA4_F_.

### Enzyme characterization

**Figure 5:**
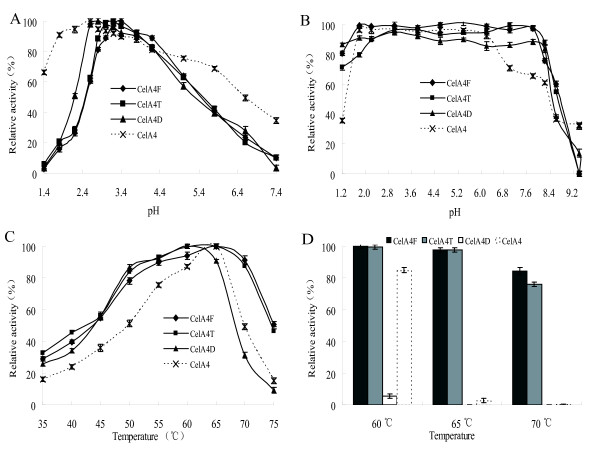
**Characterization of purified CelA4_F_, CelA4_T_, CelA4_D_, and native CelA4**. A: Effect of pH on β-1,4-glucanase activity. Activity assays were performed at 65°C for CelA4_F _and CelA4_T_, and at 60°C for CelA4_D_, in buffers with pH values of 1.2 to 7.6. B: Effect of pH on stability. After incubating each enzyme at 37°C for 1 h in buffers with pH values between 1.2 and 9.2, activities were measured in 0.1 M citric acid-Na_2_HPO_4 _(pH 3.4, 65°C for CelA4_F _and CelA4_T_, and pH 2.6, 60°C for CelA4_D_). C: Effect of temperature on β-1,4-glucanase activity measured in 0.1 M citric acid-Na_2_HPO_4 _(pH 3.4 for CelA4_F _and CelA4_T_, and pH 2.6 for CelA4_D_). D: Thermostability assay. Each enzyme was incubated at 60, 65, or 70°C in 0.1 M citric acid-Na_2_HPO_4 _(pH 3.4 for CelA4_F _and CelA4_T_, and pH 2.6 for CelA4_D_) for 1 h, and its activity was then measured under optimum conditions.

The physical properties of truncated CelA4_F_, artificially truncated CelA4_T_, deglycosylated and truncated CelA4_D_, and CelA4 [7] that affect enzyme activity were characterized and compared. CelA4_F_, CelA4_T_, and CelA4_D _showed optimum activity at pH 3.4, as opposed to pH 2.6 for native CelA4. The first three enzymes were less active between pH 1.2 and 2.2 and between 4.8 and 7.6 than was native CelA4 (Figure 5A). CelA4_F_, CelA4_T_, CelA4_D_, and CelA4 were all stable between pH 1.8 and 8.2, at 37°C for 1 h, but the first three enzymes were more stable at pH 1.2 (>70% retention of activity) than was CelA4 (~36% retention of activity) (Figure 5B). All of the enzymes displayed maximum activity between 60 and 65°C (Figure 5C). Notably, CelA4_F _and CelA4_T _were relatively stable at 75°C, as they maintained > 75% of their initial activities after a 1-h incubation at pH 3.4, 75°C. Under the same conditions, CelA4_D _and CelA4 lost all activity (Figure 5D).

CelA4_F_, CelA4_T_, and CelA4_D _were highly resistant to the acidic and neutral proteases, including trypsin, α-chymotrypsin, collagenase, pepsin, and subtilisin A, and they retained more than 70% of their activities after incubation with these proteases at 37°C for 2 h. This resistance to acidic and neutral proteolysis had been found previously for CelA4 [7].

### Nucleotide sequence accession number

The nucleotide sequence of the β-1,4-glucanase gene (*CelA4*) from *Alicyclobacillus *sp. A4 was deposited in GenBank under the accession number GU576556.

## Discussion

We previously isolated an extremely acidic GH 51 β-glucanase, CelA4, from thermoacidophilic *Alicyclobacillus *sp. A4. Herein, we described the gene sequence and the expression of *CelA4*_*F*_, which encodes CelA4 lacking the N-terminal signal sequence. Its deduced amino acid sequence is only 42% identical to that of CelB from *A. acidocaldarius *[6], indicating that *CelA4 *is a previously uncharacterized gene. The deduced C-terminal 217-residue sequence of *CelA4 *is threonine rich (21.2%), which is a much higher than found on average (5.74%) for proteins [17]. Threonine-rich regions have been reported to be involved in fibronectin binding [18], unidirectional transport of a mineralocorticoid receptor into the nucleus [19], vanadate resistance [20], and resistance to HIV by binding to a specific receptor [21]. Structural analysis has indicated that the functions of threonine-rich regions are usually associated with those of linker regions and O-linked glycosylation [22,23]. We have found that the threonine-rich region containing the putative linker of native CelA4 and CelA4_F _was removed during secretion from *Alicyclobacillus *sp. A4 and *P. pastoris*, respectively. Therefore, the threonine-rich region may act as a molecular chaperone and be involved in proper folding of the catalytic domain. The linker has been reported to be necessary for thermostability [24,25]. Because it is removed with the threonine-rich sequence, we assume that the linker has no effect on the catalytic properties of CelA4.

Native CelA4 has excellent properties and therefore has great potential for use in industrial applications [7]. However, the yield of CelA4 was very small (0.9 U/mL) when *Alicyclobacillus *sp. A4 was cultured in a glucanase-inducing medium. For the study reported herein, we used a 3.7-L fermenter for the cultivation of *P. pastoris *containing a plasmid carrying *CelA4*_*F*_. After expression, 268 U/mL of CelA4 activity was measured in the culture supernatant, which is approximately 300-fold greater than found upon cultivation of *Alicyclobacillus *sp. A4. We also constructed a gene for the C-terminally truncated glucanase, CelA4_T _that lacked the threonine-rich region using a PCR-based gene truncation method, expressed the protein in *P. pastoris*, and obtained a yield of 420 U/mL. Expression of truncated genes has been shown to increase enzyme production and improve both activity and thermostability [26,27]. Truncated CelA4_F _and artificially truncated CelA4_T _have similar molecular masses and enzymatic properties but differ in the relative amount of enzyme produced by fermentation. This difference might be ascribed to the intracellular functions of the threonine-rich region. It is possible that the threonine-rich region acts as a molecular chaperone, but how it influences enzyme production is unknown.

Both CelA4_F _and CelA4_T _were glycosylated when expressed in *P. pastries*. It has been reported that glycosylation has significant effects on enzyme thermostability [28], the optimum pH value [29], and resistance to proteases [30,31]. CelA4_D_, CelA4_F_, and CelA4_T _had similar pH and stability profiles, but CelA4_D _was not as thermally stable as the other two proteins. For example, CelA4_D _exhibited ~30% of its maximal activity at 70°C, whereas both CelA4_F _and CelA4_T _retained ~90% of their initial activities. Moreover, CelA4_F _and CelA4_T _were thermostable at 70°C, but CelA4_D _was not stable at 60°C. Although the molecular mass of CelA4_D _was similar to that of native CelA4, these enzymes differed with respect to their pH and temperature profiles and, most notably, their stabilities. Therefore, heterologous expression of CelA4_F _increased the amount of the enzyme produced and improved the thermostability by incorporating sugar residues post-translationally.

CelA4_T _and CelA4_F _exhibited similar enzymatic properties, namely a pH optimum of 3.4, a temperature optimum of 65°C, stability between pH 2.0 and 8.2 and at 70°C, and they were both active and stabile under simulated gastric conditions. Both enzymes could decrease the viscosity of barley-soybean feed (data not shown), but more of the former was produced during *P. pastoris *fermentation. Therefore, in the future we will produce CelA4_T_, not CelA4_F_, by heterologous expression for commercial applications.

## Conclusions

For the study reported herein, we identified and cloned the β-1,4-glucanase-encoding gene (*CelA4*) found in *Alicyclobacillus *sp. A4, and achieved high-yield expression of CelA4_F _in *P. pastoris*. CelA4_F _was truncated and glycosylated during fermentation. Expression of CelA4_T_, which lacks the threonine-rich region, produced greater amounts of protein and had glucanase activity identical to that of CelA4_F_. We speculate that the threonine-rich region might act as a molecular chaperone that ensures proper folding of the catalytic domain. Glycosylation was necessary for the thermostability of both CelA4_F _and CelA4_T_. All of our data indicate that recombinant CelA4_T _produced using a *P. pastoris *fermentation system will have great potential as a β-1,4-glucanase for use in the feed industry.

## Methods

### Strains, plasmids, and chemicals

The strain *Alicyclobacillus *sp. A4 was deposited in the China General Microbiological Culture Collection Center under the registration number CGMCC3147 [32]. *Escherichia coli *JM109 was obtained from TaKaRa (Dalian, China). *P. pastoris *GS115 and the pPIC9 vector were obtained from Invitrogen (San Diego, CA, USA). Barley β-glucan was supplied by Sigma (St. Louis, MO, USA). T4 DNA ligase and restriction endonucleases were obtained from Promega (Madison, WI, USA). All other chemicals were of analytical grade and commercially available.

### Cloning and expression of the β-1,4-glucanase gene (CelA4_F_)

Using the sequences of an N-terminal peptide and five internal peptides of native CelA4 [7], the full-length coding gene for CelA4 (*CelA4*) was identified in the genome of *Alicyclobacillus *sp. A4 using BLASTp (Sequencing of the complete *Alicyclobacillus *sp. A4 genome is in progress). The sequence of the N-terminal signal peptide was predicted using SignalP http://www.cbs.dtu.dk/services/SignalP/. Alignment of multiple protein sequences was accomplished using ClustalW [33]. Vector NTI 10.0 software was used to identify homologous and identical residues after sequence alignment and to predict the molecular mass of the mature protein.

To construct the plasmid containing *CelA4*_*F*_, the gene encoding CelA4_F _was PCR-amplified using the primers CelA4_F_F: GCA**TACGTA**ATGGAGGCGACTATGCAAGCAGC and CelA4_F_R: GAA**GCGGCCGC**TCAGACACCCACAAAATGAGAAACCAC (*SnaB*I and *Not*I sites are bold) with *Alicyclobacillus *sp. A4 genomic DNA as the template. The PCR-amplified gene fragment was cloned in-frame at the downstream site of the α-factor (signal peptide) present in the pPIC9 vector and transformed into *P. pastoris *competent cells. Positive transformants were cultured in minimal dextrose medium or minimal methanol medium, and the culture supernatants were screened for glucanase activity.

### High-cell-density fermentation of recombinant CelA4_F_

The positive transformant with the highest level of β-1,4-glucanase activity was grown in a 3.7-L fermenter (Bioengineering KLF 2000, Wald, Switzerland) using a four-step fermentation strategy [13] that was scaled up. The *Pichia *fermentation was performed according to instructions obtained from Invitrogen. Fermentation began with a batch growth phase in 2.0-L basic sodium medium and the following conditions were used: agitation speed, 1,000 rpm; ventilation rate, 1.6 vvm; temperature, 30°C; pH, 4.5 (adjusted with 6 M NH_4_OH). The first phase was terminated when the glycerol in the basic sodium medium was consumed completely (about 18 h) and the cell mass had increased to > 100 g/L (wet cell weight). The second glycerol (50%, w/v) fed-batch phase was initiated at 70 mL/h for ~6 h to further increase the cell mass to ~170 g/L. During the third phrase, a glycerol/methanol (8:1, v:v) mixture was fed at 25 mL/h for ~4 h to transition the culture from glycerol to methanol. In the fourth phase, methanol was fed at 6-7 mL/h for ~156 h. During the fermentation process, dissolved oxygen was kept above 20%. Culture samples were collected every 12 h and subjected to cell mass and enzyme activity analyses.

### Purification and identification of recombinant CelA4_F_

The culture supernatant (about 2,500 mL) was concentrated ~15-fold by ultrafiltration using hollow-fiber membranes with molecular weight cut-offs of first 6 kDa (Motimo, Tianjin, China) and then 5 kDa (Vivascience, Hannover, Germany). The resulting solution was dialyzed against 20 mM McIlvaine buffer (0.2 M Na_2_HPO_4 _containing 0.1 M citric acid), pH 7.5, and loaded onto a HiTrap Q Sepharose XL FPLC column (Amersham Pharmacia Biotech, Uppsala, Sweden) equilibrated with the same buffer. Proteins were eluted using a linear gradient of NaCl (0-1.0 M) in the same buffer. Fractions with enzyme activity were collected and subjected to SDS-PAGE [34]. To identify the purified protein as CelA4_F_, the protein band was excised from the gel, digested with trypsin, and sequenced using liquid chromatography/electrospray ionization tandem mass spectrometry (MALDI-TOF-MS/MS) at the Institute of Zoology, Chinese Academy of Sciences.

### Cloning of the artificially truncated β-1,4-glucanase gene (CelA4_T_)

According to the molecular mass comparison of CelA4_F _and native CelA4 and the sequence analysis, residues 498-715 was supposed to be hit by profiles with a high probability of occurrence (Entry: PS50325) based on ScanProsite analysis http://www.expasy.ch/tools/scanprosite. Residue 497 was predicted to be the truncation site. *CelA4*_*T *_encoding the truncated protein (residues 40-497) was PCR-amplified using the primers CelA4_T_F (GCA**TACGTA**ATGGAGGCGACTATGCAAGCAGC) and CelA4_T_R (GAA**GCGGCCGC**AAACTGTGGCCCTTGTGGCTGATGC) (*SnaB*I and *Not*I sites are bold) with genomic DNA of *Alicyclobacillus *sp. A4 as the template. CelA4_T _was expressed in *P. pastoris *and subjected to the same purification procedure as described above.

### Deglycosylation of CelA4_F_

Purified CelA4_F _(a ~2 μg) was treated with 20 U of Endo H for 2 h at 37°C according to the supplier's instructions (New England Biolabs, Ipswich, MA, USA) and then analyzed by SDS-PAGE.

### Enzyme assay

All enzymatic assays were performed in triplicate. The final reaction systems contained 50 μL of an appropriately diluted enzyme and a 450-μL solution containing 1% barley β-glucan (w/v) and 200 mM McIlvaine buffer at the optimum pH previously determined for each enzyme. Reactions were allowed to proceed for 10 min at the optimum temperatures and then terminated by adding 1.5 mL dinitrosalicylic acid [35]. Each mixture was heated in a boiling water bath for 5 min. After addition of 1.0 mL H_2_O, the absorbance of each mixture at 540 nm was measured. One unit of enzyme activity was defined as the amount of enzyme that catalyzed the formation of 1 μmol glucose per minute.

### Biochemical characterization of purified CelA4_F_, CelA4_T_, and CelA4_D_

The pH optima for the enzyme activities of CelA4_F _and CelA4_T _were determined at 65°C and at 60°C for CelA4_D_. The enzymes were incubated for 1 h at 37°C in the absence of substrate, and then their activities were measured at pH 3.4 and 65°C for CelA4_F _and CelA4_T_, and at pH 2.6 and 60°C for CelA4_D_. The buffers used were 0.1 M KCl-HCl for pH 1.2-2.2, 0.1 M McIlvaine buffer for pH 2.6-7.6, 0.1 M Tris-HCl for pH 8.0-9.0, and 0.1 M glycine-NaOH for pH 9.4-12.0. The optimal temperature for enzyme activity was determined using the McIlvaine buffer (pH 3.4 for CelA4_F _and CelA4_T_; pH 2.6 for CelA4_D_) at temperatures between 35°C and 75°C. The thermostability of each enzyme was determined by incubating the enzyme (100 μg/mL) in McIlvaine buffer (pH 3.4 for CelA4_F _and CelA4_T_; pH 2.6, for CelA4_D_) at 60°C, 65°C, or 70°C without substrate for 1 h and then measuring the enzyme activity under optimum conditions.

To examine resistance to different proteases, purified CelA4_F_, CelA4_T_, and CelA4_D _(2 μg/mL) were each incubated with 200 μg/mL trypsin, α-chymotrypsin, collagenase, pepsin, or 500 μg/mL subtilisin A at 37°C and at the pH optimum of the specific protease for various lengths of time. Incubations in the absence of each protease served as the controls. Activities were determined under the standard assay conditions of pH 3.4, 65°C for CelA4_F _and CelA4_T_, and of pH 2.6, 60°C for CelA4_D_.

## Abbreviations

Endo H: endo-β-*N*-acetylglucosaminidase H; SDS-PAGE: sodium dodecyl sulfate-polyacrylamide gel electrophoresis.

## Competing interests

The authors declare that they have no competing interests.

## Authors' contributions

YB participated in the design of the study, the fermentation development, data analysis, and in the writing of the manuscript. JW participated in the construction of the recombinant plasmids. CL participated in the fermentation procedures and data analysis. YF participated in the biochemical characterization of the enzymes. ZZ participated in the writing and reviewing of the manuscript. BY participated in the writing and editorial supervision of the manuscript. PS supervised the experiments. HH participated in the gene sequence analysis. HL participated in the result analysis and the writing of the manuscript. All authors read and approved the final manuscript.
